# The Acute Effects of Cognitively Demanding Physical Activity on Inhibitory and Affective Responses in Children: An Online-Based Mixed Methods Approach

**DOI:** 10.3390/children9121896

**Published:** 2022-12-02

**Authors:** Ricardo M. G. Martins, Emiliano Mazzoli, Michael J. Duncan, Cain C. T. Clark, Emma L. J. Eyre

**Affiliations:** 1 Centre for Sport, Exercise and Life Sciences, Coventry University, Coventry CV1 5FB, UK; 2 Institute for Physical Activity and Nutrition, School of Exercise and Nutrition Sciences, Faculty of Health, Deakin University, Geelong, VIC 3216, Australia

**Keywords:** children, physical activity, executive function, enjoyment, affective responses, focus groups

## Abstract

This online study investigated the acute effects of a cognitively demanding physical activity (CDPA) vs a simple physical activity (SPA) bout on children’s inhibitory and affective responses. Using a counterbalanced within-subjects’ crossover design, thirty-nine participants aged 9–12 years old (29 boys; M_age_ = 11 ± 1 years) performed a CDPA and a SPA bout online (via ZOOM) for 15 min. Inhibition (Stroop test) was measured at the baseline, 1 and 30 min following the physical activity (PA) bouts, and self-report measures of affect, mental and physical exertion were taken prior, during and post-PA. Additionally, 31 children took part in semi-structured focus groups to explore the factors affecting their enjoyment. The quantitative results suggest no significant differences on inhibitory responses, affect and physical exertion (all *p >* 0.05). However, the CDPA induced more mental exertion than the SPA did (*p <* 0.05). In the focus groups, four themes were identified: physical exertion (e.g., tiredness), social (e.g., teams/groups), environment (e.g., outdoors and competition) and emotional (e.g., fun/enjoyment). Some children (*n =* 18) reported that the CDPA condition confused them, and to make these activities more interesting and enjoyable, they suggested performing the activities outdoors (*n =* 15) and including other children as part of a group/team (*n =* 19). The findings suggest no additional benefit of a cognitively enriched physical activity compared to an SPA bout on the inhibitory responses, affect and enjoyment. Using the instructions provided and given the low cost, the easy administration and the minimal amount of equipment and time involved, either of the approaches may be used in a diversity of contexts (i.e., online, schools or outdoors), and it is worth exploring the effects of these conditions on other aspects of executive function.

## 1. Introduction

Executive functioning (EF) can be described as a set of cognitive processes responsible for goal-oriented behaviours [1]. These functions are commonly classified into inhibition (i.e., the ability to refrain from impulsive responses and attention), working memory (i.e., the ability to manipulate and hold information in memory) and cognitive flexibility (i.e., the ability to shift between tasks and respond appropriately to the changing demands) [1]. Physical activity (PA) has been proposed to improve EF [2,3], wellbeing, self-esteem, resilience [4] and also mental health [5,6]. These are key components of academic performance, and it is crucial that they are developed in childhood [4,7].

The reviews examining the acute effect of PA on EF suggest that PA bouts longer than ten min and shorter than thirty min, comprising of submaximal or maximal intensities, might improve EF in adults and children [3,8]. However, from the short-term effects of PA on EF, only a small number of studies on children were included in the reviews mentioned, and Hillman et al. [9] and Schmidt et al. [10] recently reported that the most effective quantitative (e.g., intensity and duration) and qualitative PA (e.g., simple or cognitively enriched) is yet to be determined. The acute effects of PA on EF in children are in demand, as these have the potential to be easily implemented in core moments of the day which would lead to improved EF, and consequently, better academic performance in schools [4,7].

Cognitively demanding physical activity (CDPA), a physical task that requires participants to respond appropriately to cognitively engaging tasks for correct execution, has been suggested to improve EF [11]. Researchers have hypostatised that CDPA might induce additional benefits on top of a simple activity as EFs are requested while moving, and the cognitive and coordinative complexity of the movements induce extra neural stimulation [12,13,14]. The studies that investigated the acute effects of CDPA on EF are limited, when considering children of younger ages (6–18 years), some authors have found positive effects on attention and EF [11,15,16,17,18], while others have found null [19,20,21] or even negative [22] effects of CDPA on EF. Collectively, these studies employed different quantitative designs (e.g., between and within subjects), durations (e.g., 10 to 42 min), cognitive tests (e.g., attention, memory recall and EF) and mental engagement, leading to a situation where there is no clear evidence of the additional benefit of CDPA compared to a simple PA bout, and more studies are needed exploring specific EF domains. Besides the hypothesised benefit of CDPA compared to a simple PA, understanding the children’s affective responses to CDPA might also help to provide recommendations for CDPA use, as enjoyment and feelings can be associated with EF performance, participation and adherence to PA [23].

Affective responses and enjoyment have been studied quantitively and qualitatively for other types of PA, and they are known to be a critical aspect of PA engagement, participation, adherence and even positive cognitive responses [24,25]. However, the current problem is that CDPA research has not yet considered how children perceive these mental engaging activities, therefore, exploring their responses in depth is needed. The widely known constituents of the self-determination model (i.e., psychological perception of satisfaction), competence, autonomy and relatedness might play an important role in enjoyment and engagement in these activities [26]. Therefore, addressing this gap can expand our knowledge of what children perceive, like and dislike while they are performing CDPA bouts, informing and creating more robust and ecological approaches that inform researchers and teachers how to create more enjoyable activities [5,6]

Children’s patterns of movement are usually highly intermittent [27], comprising various PA bouts of low to high intensities, which is their normal way of moving and playing [28]. However, evidence of the acute effects of CDPA on EF using normal patterns of movement (e.g., running, jumping, walking, throwing, etc.) is scarce. To date, the findings seem to be equivocal, with some authors reporting positive [11], null [20] and even negative effects [22]. These studies were applied in physical education classes, classroom-based PA, and the duration (e.g., 20–42 min) varied between the protocols, leading to more research being required to ascertain conclusions. In addition to this, creating an approach that could be easily implemented in various contexts (i.e., online, schools or outdoors) based on the children’s natural movement patterns might help improve EF, tackle sedentarism and increase their motivation towards participation.

The amount of sedentary behaviour and recreational screen time has raised among young children during the COVID-19 pandemic [29,30,31]. This is a particular concern, since the COVID-19 pandemic, children’s PA levels reduced significantly (−10.8 min/day and −91 min/day) [32,33], and also decreased subjective wellbeing compared to previous years [34], raising concerns about the pandemic’s impact on children’s physical and mental health [32,33]. Consequently, as online PA approaches have been shown to reduce sedentary time [35], the use of this technology (e.g., videos, apps and other digital technologies) can be easily and successfully integrated across contexts such as in schools’ physical education classes [36], sports clubs or home environments [37,38]. Additionally, online interventions can effectively promote PA and affective responses in children [39]. Thus, an online approach developed by specialists could be easily implemented and given in a wide variety of environments by teachers, parents and practitioners with little to no experience delivering these activities. On top of this, it would help avoid the teachers’ burden without requiring a specialist while reaching many children, thereby increasing their PA levels and potentially improving their EF.

Given the lack of studies examining the effects of CDPA on EF and affective responses, this study aimed to use a mixed-method approach to investigate two distinct types of online PA bouts (SPA vs CDPA) with similar intensity on their inhibitory responses, affect, perceived exertion and arousal. It was hypothesised that CDPA, due to the combination of PA and cognitive stimulation, is more effective than a simple activity without cognitive demands is. The findings might inform how to better design activities in the future to create adherence and more enjoyable experiences while eliciting the same physical health and/or cognitive benefits.

## 2. Materials and Methods

### 2.1. Participants

Thirty-nine participants (9–12 years old: 29 boys; M_age_ = 11 ± 1 years) were recruited from a handball club (*n* = 10) and a public school (*n* = 19) (participants in school sports) in Porto, Portugal and a football club (*n* = 10) in West Midlands, UK (i.e., convenience sampling). We considered the cognitive outcomes for a within–between interaction for a repeated measures ANOVA as the previous literature suggests small to moderate effects of PA on EF in children [2,3,9,40]. The minimum number of participants required to detect significant differences was 28, and this value was based on an a priori power calculation conducted using G-power software (Power = 0.8 and α = 0.05; ES(f) = 0.14–0.25 or η^2^p = 0.02−0.06) [41]. The participants with musculoskeletal, cognitive impairments (e.g., intellectual disability), mental health disorders (e.g., depression), cardiovascular contraindications to PA or those taking any medication for blood pressure or cardiac conditions were excluded (information obtained through the PA readiness questionnaire). This research received ethical approval from Coventry University (P114425).

### 2.2. Protocol

**Figure 1:**
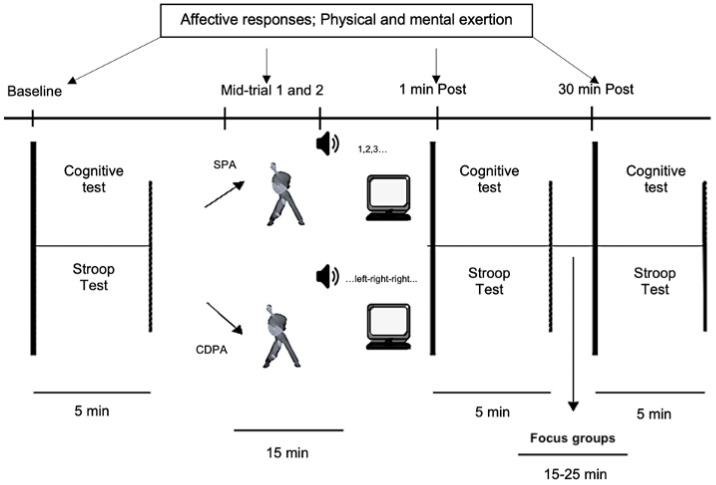
The time course and procedure for the SPA and CDPA conditions.

The quantitative protocol used in the current study employed a counterbalanced within-subjects crossover design, where the participants were randomly allocated to a sequence involving SPA and CDPA (www.randomizer.org, accessed on 15 October 2019). Due to the COVID-19 pandemic, all of the data collection was conducted via ZOOM (Zoom Video Communications, Inc., San Jose, CA, USA) when the leading researcher was on call with the participants. The participants were instructed to stand in front of their own laptops (i.e., a requirement to participate) in a clear area (i.e., where they could do the movements safely), and they were instructed to follow verbal and written instructions. All of the participants completed two experimental conditions (i.e., SPA and CDPA) on two different days (1–1.5h duration). Each condition lasted for 15 min (see Figure 1), which is representative of the regular UK school recess time, and the dose recommended suggests PA bouts that are between 10-20 min for greater benefit [3,8]. To ensure the conditions were intensity matched, both of the conditions employed the same number of movements, and the commands (verbal stimuli) were the only variation between them (Section 2.5 for more details). The System for Observing Fitness Instruction Time (SOFIT) analysis was used to estimate the energy expenditure in both of the conditions (1.75–1.94 Kcal/condition were estimated), and both of the conditions fell into the category of moderate-intensity PA. This calculation was based on the observation of the condition videos, and to ensure reliability, at least 15% of it was coded simultaneously by two independent observers (ICC = 0.90). These measures and procedures have been reported to provide a valid estimated energy expenditure for children [42,43].

Before starting the trial, children performed from four to six repetitions of each movement involved in the experimental conditions. The instructions on how to perform the cognitive test and affective scales were provided (please see *quantitative assessment* for more details). To avoid learning effects (improvement due to the practice), the participants were required to perform between at least two and four complete trials of the cognitive test on their first day. These effects have been investigated, and they were diminished by the repeated exposure to the task, with no significant differences found among the 2nd–4th administrations [44,45,46]. Additionally, the affect scales were explained and presented, where the participants had the chance to practice before the trials. This was allowed to ensure they were familiar with the experimental processes, and the lead researcher administered the tests and scales to assure homogeneity. The affective scales were collected at the baseline, during (coded as ***mid-trial 1*** and ***mid-trial 2*** (following exercises 1 and 2 and 3 and 4, respectively), 1 min post (following exercises 5 and 6) and 30 min post (please see next section for more details). The inhibitory data were collected at the baseline, 1 min and 30 min post (further information in the next section).

The participants were presented with a pre-recorded animation video demonstrating the movement sequence to be performed. This ensured that the participants received consistent instructions and had a visual reference to follow and complete the movements accurately. The videos were presented via ZOOM and in the participants’ mother language (i.e., English/Portuguese). The children’s execution was recorded and later analysed to assess the accuracy of movement performance using a fidelity checklist (see Appendix A). 

### 2.3. SPA

The participants undertook an intermittent movement sequence based on functional movement skills that are widely used in children while they are moving and playing in schools [47,48]. The following movements were included in the sequence: (1) 30 jumps; (2) run on the spot (2 min), (3) squat and kick, (4) windmill (stand and touch the right foot with the left hand and vice versa) (30 rep), (5) high step march (30 rep) and (6) 30 ski hops/jumps. The overall protocol lasted 15 min, including the instructions and scale administration. 30 s rest for each activity (i.e., 1–2 min each bout) was implemented, which is in line with the previous literature [8].

### 2.4. CDPA

**Figure 2:**
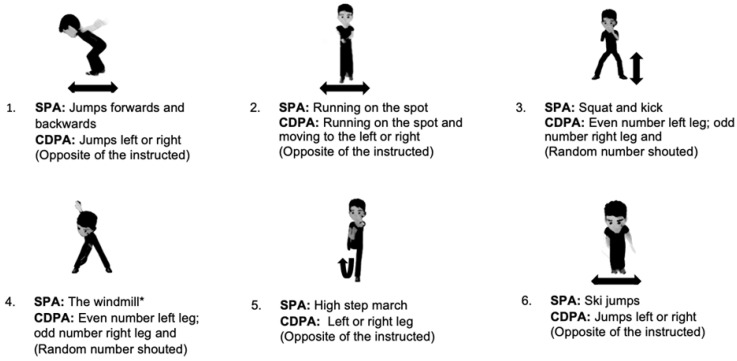
Visual representation of the SPA exercises/movements presented to the participants.

The activities performed for this condition were the same as in the simple condition except that the commands were modified to increase the cognitive demand of the task while maintaining the intensity and duration stability. For activities 1, 2, 5 and 6 (see Figure 2), the participants were asked to move in the opposite direction of what was instructed (e.g., when they were required to move/jump to the left, they would move/jump to the right), for activities 3 and 4 (see Figure 2), a random number was shouted, and they would move the left leg first if the number was odd, and they would move the right leg first if it was even. Changes to the level of coordination complexity and cognitive demand were designed as previously recommended by Tomporowski et al. [7], and these included at least one of the following demands: (1) Cognitive interference: introducing random changes in the conditions under which the task is performed (e.g., an unpredictable component that requires cognitive engagement and forces adaptation). (2) Trigger core EF: including a task/component that relies directly on a specific core EF (working memory, inhibition and/or cognitive flexibility). 

### 2.5. Quantitative Assessment 

#### 2.5.1. Inhibitory Control 

**Figure 3:**
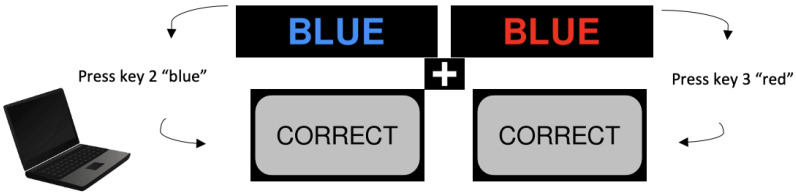
Computerised Stroop test (PsyToolkit) visual representation.

The Stroop test was used to assess the children’s inhibitory control. The test has been widely used in studies investigating the effects of PA on the inhibition in children [49,50,51,52]. The test was administered using PsyToolkit [53,54]: an online coding tool that allowed us to design and deliver the cognitive tests entirely online. The test presents 60 pseudo-random visual stimuli, of which 30 of them were congruent (i.e., yellow written in yellow, involving well-learned reading processes) and 30 of them were incongruent (i.e., green written in red, involving cognitive control mechanisms). The participants were asked to identify the colour of each word using the keyboard (1,2,3,4) to identify the colours (green, blue, red and yellow) and answer as quickly and accurately as possible. A fixation cross of 300 ms between the stimuli and a fixation time of 1000 ms per stimuli was used. To avoid the participant being distracted or guessing, if the participant’s reaction time was longer than 3000 ms (too slow) or faster than 200 ms (too fast), they would be excluded from the analyses [55] (see Figure 3). The total average time per response (reaction time) for the congruent and incongruent stimuli, the accuracy of responses and the interference scores (computed as incongruent-congruent) were recorded as a measure of performance. As the test was adapted to Portuguese, a pilot test including nine children was conducted before this study, and to compare the two measurements techniques, a Bland and Altman’s analysis [56] was conducted, showing that there was no consistent bias in one approach versus the other (Bias = 14.5; 95% CI (−59.4, 88.4)) and an interclass correlation (ICC) of 0.77–0.81. As the current study was conducted online, a hyperlink with automatic instructions was sent to the participants (please see Appendix for instructions). They were advised to be seated, with their hands on the keyboard and in a quiet area of the house. If the participants had vision impairments, they were instructed to wear glasses or contact lenses accordingly to their medical recommendations. The previous research suggests that this test has good reliability (ICC > 0.80) and is valid for use with children [57,58,59,60].

#### 2.5.2. Perceptions of Affect and Physical and Mental Exertion 

The self-report feeling scale (FS) [61] was employed to understand the variation in feelings. This scale rated the participants’ feelings on an 11-point bipolar scale ranging from −5 (*feeling very bad*) to +5 (*feeling very good*). Another common variation is the level of activation/arousal (i.e., excitement/relaxation, anxiety/boredom, or anger/calmness) that was experienced. A self-report felt arousal scale (FAS) [62] was used to understand these variations. This is a 6-point scale ranging from 1 (*low arousal*) to 6 (*high arousal*). FS and FAS are commonly used, and they have correlations ranging from 0.41 to 0.59 and 0.47 to 0.65, respectively, with the Affect Grid (self-reporting scale included affect and arousal) [63] showing convergent validity as these measures are theoretically related [64]. 

To measure the physical exertion, the children’s OMNI walk/run scale of perceived exertion (category range = 0–10) [65] was employed, which has a range of numbers that are familiar to children and uses age-appropriate verbal expressions as descriptors of effort. The anchors range from “*not tired at all*” (0) to “*very, very tired*” (10). This scale has been previously validated for children [65], and a metanalysis showed a good average correlation for HR (r = 0.80) and VO_2 MAX_ (*r* = 0.82) [66]. Similarly, a self-report pictorial scale for mental exertion with a range of 0-10 was used across both of the conditions, where children indicated their mental exertion during the conditions. This scale has been previously used in studies on children measuring CDPA [18,67].

### 2.6. Qualitative Approach

#### Focus Groups 

Using a convergent parallel design and criterial purposive sampling [68], the participants were grouped, which was a process that was guided by their teachers/coaches, on their age, enthusiasm and ability to communicate their experiences and opinions to ensure that a variety of views could be represented [69]. The Standards for Reporting Qualitative Research (SRQR) and the consolidated criteria for reporting qualitative research (COREQ) were used to ensure that we used an explicit and comprehensive reporting process [70]. Triangulation was used to establish credibility and contribute to the study’s trustworthiness [71,72]. In order to be transparent about the possible interpretive bias, during the interview, the analytical process and results reporting reflexivity and critical discussions were conducted to debate the themes and the study’s rigour [73,74]. When no new data emerged, or when the identification of new themes was not achieved, we considered that we had reached data saturation [30]. This enabled a broad flexible approach to analysing the data collected to produce an enriched and detailed account of the findings [75]. Following the completion of both of the experimental conditions, the participants were invited to participate in audio-recorded focus group interviews which were conducted online via ZOOM (see Table 1 for the interview guide) following the trial. The leading researcher conducted ten semi-structured interviews (Sex: Male; Occupation: PhD student) involving 31 participants. Most of the groups had 3–6 children, and only two children underwent one-on-one interviews due to difficulties in identifying a slot suitable in the children’s availability. The children were informed that this research sought to obtain their views on the different PA bouts that they performed. 

### 2.7. Data Analyses

#### 2.7.1. Quantitative 

All of the data were tested for normality and homogeneity of variance using Shapiro–Wilks and visually tested for normality using histograms, Q-Q plots, skewness and kurtosis values, where the values were within the recommended range for skewness and kurtosis [77,78]. The cognitive data have shown a normal distribution, and these measures were analysed using SPSS Statistics (v.280.0, IBM Inc., New York, NY, USA) performing 2 (Condition: Simple PA and CDPA) by 2 (Time: post-pre and post 30-pre) repeated measure analyses of covariance (ANCOVA) which were adjusted for age, sex and the number/accuracy of the movements. If the covariates were not significant, they were excluded from the model, and repeated measures analyses of variance were performed (ANOVA). Where sphericity was violated, Greenhouse–Geisser was used to adjust the degrees of freedom, and these are reported. If significant effects were found (*p* ≤ 0.05), follow-up post hoc tests, with Bonferroni adjustments where they were applicable, were conducted to discern the differences between the conditions. If they were not appropriate (due to the number of comparisons), LSD follow-up post hoc tests were used as the criteria of K = 3 groups or time points was met [79]. The effect sizes were calculated to understand the magnitude of the mean differences, and these were interpreted using as 0.01 (small), 0.06 (medium) and 0.14 (large) using partial eta-squared η^2^p [80].

As the effect and perception of the physical and mental exertion data did not meet the normality criteria, a non-parametric repeated measures ANOVA (Friedman) using Jamovi (V.1.2.17) was employed to analyse the affect scales and pairwise comparisons. Durbin Conover equations were used to discern the differences across the time points, and the results of these analyses are provided by medians (Med) and interquartile ranges (IQR). A non-parametric two-related samples *t*-test was employed to discern the differences across the conditions regarding the number and accuracy of the movements.

#### 2.7.2. Qualitative 

All of the interviews were recorded via ZOOM and verbatim transcribed into Microsoft Word (Microsoft, Redmond, WA, USA). The interviews were conducted in Portuguese and English. The transcriptions in Portuguese were translated into English by a native Portuguese speaker, and they were discussed with a native English speaker before being analysed. Following an inductive analysis of the transcriptions, Braun and Clarke [75] procedures (familiarisation, read and rereading, coding, categorisation, reviewing themes and defining and naming themes) were employed, creating themes, sub-themes and quotes. Following this analysis, pen profiles were constructed, a technique that is used to present interactions outcomes via diagrams of composite key emergent themes, and they are considered to be appropriate and accessible to researchers with an affinity for both qualitative and quantitative backgrounds [81,82]. 

## 3. Results

The distribution of the cognitive data following a Shapiro–Wilk test did not show evidence of non-normality (W ≥ 0.66, *p >* 0.05). In contrast, the effect and the physical and mental exertion data departed significantly from the normality data (W ≥ 0.41, *p* ≤ 0.05). The results from the ANCOVA models showed that the session order, age, sex, context (i.e., schools vs. clubs or Portugal vs. the UK) and the number/accuracy of movements executed during the trial were not significant covariates of the effects of the PA bouts on EF (all *p*-values > 0.05), except for the incongruent stimuli, reported below). However, the movement accuracy was statistically significantly lower for the CDPA than it was for the SPA (*p* = 0.004).

### 3.1. Inhibitory Assessment 

#### Stroop Test

**Figure 4:**
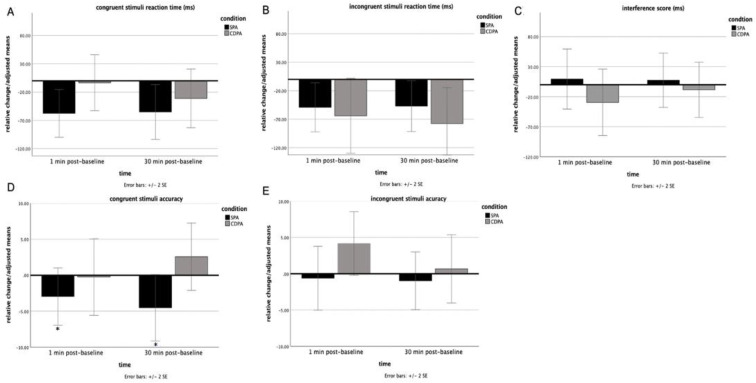
Effects of SPA and CDPA on (**A**) congruent reaction time, (**B**) incongruent reaction time, (**C**) interference, (**D**) congruent accuracy, and (**E**) incongruent accuracy. The plots are presented with relative change/adjusted means in ms and 95% CI. * Significant at *p <* 0.05 (condition effect). The percentage of error rate represents the accuracy.

There were no significant effects between the conditions for the congruent (main effect: F (1,38) =.99, *p* = 0.33, η^2^p = 0.025), incongruent (main effect: F (1,37) =.21, *p* = 0.65, η^2^p = 0.006 sex was a significant covariate (*p* = 0.039)) and interference (main effect: F (2,76) = 0.58, *p* = 0.56, η^2^p = 0.015) scores of the Stroop test (see Table 2 and Figure 4). However, the SPA condition had a higher accuracy for the congruent stimuli compared to the CDPA one (condition effect: F (1,37) = 4.2, *p* = 0.046, η^2^p = 0.103 mean diff = 4.9; main effect: F (1,37) = 1.8, *p* = 0.187, η^2^p = 0.046). The accuracy for the incongruent stimuli did not have any statistical differences across the conditions (main effect: F (1,33) = 0.99, *p* = 0.33, η^2^p = 0.03).

### 3.2. Affect Scales 

A Friedman model revealed the statistically significant differences in the FS scores χ^2^_(8)_ = 28.5, *p* = 0.001 within the conditions (see Table 3). The participants from the SPA condition reported a lower pleasure value at 30 min after compared to the values at baseline and at mid-trial 1 (*p <* 0.05) and higher values of pleasure than at 1 min post and at mid-trial 2 (*p <* 0.05). For the CDPA condition, the participants reported lower values of pleasure at mid-trial 2 compared to the baseline (*p* = 0.018) and also at mid-trial 2 when compared to mid-trial 1 (*p* = 0.015). However, the participants reported higher pleasure values at 30 min post than they did at mid-trial 2 (*p* = 0.001). No differences in the FS were found when we were comparing both of the conditions (all *p >* 0.05). The FAS was statistically significant across the conditions χ^2^_(9)_ = 78.4, *p* = 0.001. The participants during the SPA reported higher values of arousal at mid-trial 1, mid-trial 2, 1 and 30 min post when compared to the baseline (all *p >* 0.05). At 30 min post, the participants reported higher values than they did at mid-trial 2 and at 1 min after (all *p >* 0.05). In the CDPA condition, the participants reported higher values of arousal at mid-trial 2, 1 and 30 min post compared to those at the baseline (all *p >* 0.05). However, the participants reported lower values of arousal at mid-trial 1 compared to baseline (*p* = 0.001) and higher values 30 min post compared to 1 min post (*p* = 0.042). There were no statistically significant changes between the conditions (all *p >* 0.05). 

### 3.3. Physical and Mental Exertion

Statistically significant differences were found for the OMNI scale χ^2^_(9)_ = 97.1, *p* = 0.001. Within the conditions, the participants perceived higher physical exertion values when the PA bout started and until it finished (all *p <* 0.05), but at 30 min post, the participants reported significantly lower values compared to 1 min post-PA. However, for the SPA condition, there were statistically significant differences at 1 min post compared to at 30 min post, where the participants reported lower values of physical exertion at 30 min following the PA bout. Additionally, the participants reported higher values at mid-trial 2 and 1 min post compared to mid-trial 1 (all *p <* 0.05). There were no statistical differences between the conditions (all *p >* 0.05). 

The mental exertion scale was statistically significant between the conditions χ^2^_(5)_ = 69.3, *p* = 0.001. Comparing SPA to CDPA, the cognitive condition induced higher values of mental exertion at all of the time points compared to SPA (all *p <* 0.05). Within the conditions, the CDPA condition reported higher values of exertion mid-trial 2 compared to mid-trial 1 (*p* = 0.008). 

### 3.4. Perception of the Physical Activity Bouts

**Figure 5:**
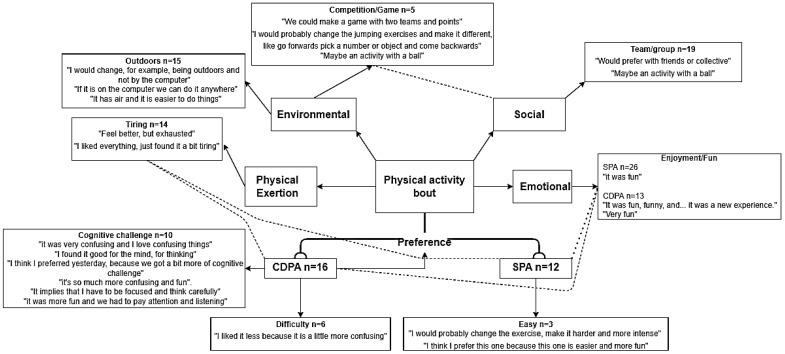
Diagram of children’s perceptions of the PA conditions. ------ stands for similarities.

The participant’s perceptions of the PA bouts were convergent and divergent between the conditions. As the pen profile suggests (see Figure 5), the participants described both of the activities similarly in terms of enjoyment and tiredness in the convergent themes. The children explained, “it was fun” [F10], and “I liked everything, just found it a bit tiring” [M10]. Regarding the divergent themes between the conditions, the participants perceived the CDPA condition as being cognitively exigent. Some of these views differ among the participants, e.g., some of them viewed the cognitive challenge positively: “It was very confusing, but I love confusing things” [F11] and “It was so much more confusing and fun” [F10]. On the other hand, some of them have reported it as a negative aspect of the activity, suggesting this might have been hard to execute “I liked it less because it is a little more confusing” [M10]. Therefore, this shows that the participants might have different personality traits or a lack of motivation, and thus, they perceive mental challenges differently.

Although both of the conditions were reported as being fun, as represented in the pen profile (Figure 5), more of the participants associated this word with the SPA condition. However, their preference for the conditions was similar, leading to a global conclusion of enjoyment and tiredness for both of the conditions. Furthermore, the SPA condition was perceived as being easier due to the facility of executing the movements.

The participants suggested various ways to modify the activities (mainly relating to the environmental and social themes) to make them more enjoyable. Most of the children suggested activities outdoors, “I would change for example being outdoors” [M11] and it being conducted in a more social environment “would prefer it with friends” [M10]. In contrast, some of them thought that the online modality was accessible “we can do it anywhere” [M11], while others expressed their preference for activities that involve competition/games “we could make a game with two teams and points” [F11] and they proposed an “…activity with a ball” [F11]. 

## 4. Discussion

This study investigated the acute effects of SPA vs CDPA bouts on the inhibitory and affective responses in children, and these were matched for intensity and duration, and they only differed in the level of mental complexity. The present study addresses a noted gap in the literature by examining and exploring how CDPA influences the inhibitory and affective responses using an online mixed-method approach. The results showed no additional benefits of the CDPA condition compared to the SPA one on the inhibitory and affective responses. However, the children’s performance at the congruent stimuli of the Stroop task was less accurate following the CDPA condition than in the SPA one. Considering the participants’ perception of these bouts, during the focus groups, some of them reported confusion following the CDPA condition, which might lead to reduced accuracy on the Stroop test. Based on the combination between the observed performance and direct feedback from children, we believe that the reduced accuracy might be linked to the excessive cognitive demand of the task as out of the ten participants who reported confusion, nine of them showed a lower accuracy. Although confusion was not a key theme in the SPA data, individual differences were present as some of the children preferred the CDPA condition while others preferred the SPA one. Despite the differences in preference, the children found both activities to be fun albeit tiring. Their enjoyment could be explained by the task’s novelty and their interest in sports. This aligns with the self-determination theory [26], based on the perception of PA, where their enjoyment can be explained by the psychological perception of satisfaction, competence, autonomy and relatedness, showing the novelty of this study and representing recommendations to target interventions. 

One of the hypothesised mechanisms that is mostly believed to influence EF enhancement is arousal [4,83,84]. On top of this mechanism, an additional benefit of CDPA is expected to be that the cognitive and coordinative complexity might induce extra neural stimulation [11,12,13,14]. In our study, we matched both of the activities for intensity and duration, and both of the conditions significantly elicited their arousal state during and post-PA. The affect scales showed no significant differences between the conditions across all of the time points. Additionally, the quantitative data (mental exertion self-reporting scale) showed a difference between the conditions. The CDPA condition elicited higher values of mental exertion throughout the bout, and this is confirmed by the data obtained in the focus groups, as participants reported it to be confusing. However, our study did not find any improvement in the CDPA condition compared to the SPA one on the participants’ inhibitory responses. Previous studies have reported added benefits of CDPA on EF and attention [11,13,15,16,17,18]. However, from these studies, only two of them reported improvements following CDPA on the inhibitory responses [13,17]. Jäger et al. [17] compared CDPA to a rest control condition and Vazou and Smiley-Oyen [13] explored the effects of PA integrated with math practice and seated math practice, and both of the studies found an improvement in the inhibitory responses. Yet, these studies compared CDPA to a rest control condition rather than a PA non-cognitive condition, and therefore, it is unclear and difficult to ascertain the benefits of CDPA compared to simple PA at the same intensity. On the other hand, Benzing et al. [15], and later Egger et al. [22], compared the experimental conditions involving low and high cognitive demands, and they found no added benefit of CDPA on the inhibitory responses compared to low mental engagement conditions and a rest control condition. As the aforementioned studies employed different research designs and protocols (e.g., exergaming and classroom-based), therefore, the effects of CDPA on inhibitory responses are still inconclusive. More research is needed to elucidate the effects of CDPA not only on inhibition but also consider other EF (i.e., working memory and cognitive flexibility). 

Pesce et al. [85] suggested that the physiological and psychological aspects must be adjusted for each participant to ensure effectiveness while using CDPA. To avoid the effects of individual complexity, our study was designed based on two core aspects. Firstly, the movements were based on functional movements which are natural and ecological types of movements associated with the children’s patterns of free play and movement [47,48]. Secondly, to create a cognitive stimulus, we followed the recommendations by Tomporowski et al. [7] to design these activities and include at least one of two core concepts: cognitive interference and/or trigger core EF. Yet, all of the participants were familiarised and only progressed to data collection if they executed the movements accurately. The qualitative data suggest that the CDPA condition induced mixed feelings, where some of them reported the cognitive challenge as being beneficial because they enjoy facing challenges. In contrast, others found it difficult and reported lower enjoyment scores, preferring easier exercises. From the current sparse evidence, a good match between the task difficulty and the individual abilities is expected to produce greater enjoyment [86] that, consequently leads to positive affective responses. [23,87]. It has also been suggested that positive affective responses could mediate the relationship between acute PA and cognition [88,89]. While some participants reported confusion induced by CDPA as being negative, in general, both of the conditions elicited positive affective responses, and the participants reported enjoyment. Although all of the participants were able to perform the bouts accurately, when we were comparing both conditions, there was no cognitive benefit of the enriched PA compared to SPA. These results might have been influenced by the level of complexity of the CDPA and justify the lower accuracy of the CDPA condition. The participants might have reached a situation of cognitive overload, where the confusion and mental exertion reported by the participants affected their inhibitory performance, leading to a situation where there is no added benefit of CDPA compared to SPA. 

A further consideration is the participants’ perception of these activities in terms of enjoyment, as it might lead or not to participation and engagement in PA [24,25]. Although both conditions were positively described in the focus groups as being fun and enjoyable, the SPA was more consistently described as being fun by the participants than the CDPA was. As these experimental conditions were conducted without being part of a game or competition, it is worth pointing out that contextual differences can contribute to the relationship between PA and EF, and this might have led to a lack of motivation [90,91]. However, the affective quantitative data show no significant differences in their affective responses between the two conditions. A possible explanation for these findings is that the participants during the interviews were more focused on describing the cognitive demands inherent to the CDPA condition experienced due to its novelty. In the SPA condition, the movements that were included were more familiar to the children. Therefore, considering the Dual Mode Theory [24,25], positive affective responses are expected when moderate intensity is employed, and our study confirms this. On top of this, the research suggests that children enjoy intermittent playground activities [92,93]. The SPA and CDPA are worth exploring further on other measures of EF and as an effective tool for increasing the PA levels in school-age children as it is likely that children will engage and consequently adhere to them. Another critical aspect of their adherence is their preference for outdoor activities, groups/teams and groups or competitions as these were suggested by the participants and can be used as a recommendation whe designing research protocols or activities for children. These suggestions can be used to increase the volume of daily PA that children undergo and contribute to the benefits of engaging in regular PA (e.g., musculoskeletal and cardiorespiratory health, mental health, quality of life, and helping to prevent non-communicable diseases). 

Furthermore, the social environment of the activities might have played a key role in this study as previous research suggests the children’s enjoyment and social interaction with their peers as one of the reasons to be physically active [94]. Lubans et al. [4] suggested that PA provides an opportunity for social interaction, where it plays a role as a mechanism that might influence EF. Our study was conducted online, limiting the social interactions inherent to it. The participants in the focus groups reported a preference for activities with teams and groups. It is known that when the participants perceive the PA as enjoyable, leading to their satisfaction and competence, they are more likely to engage in the activities, and this will lead to positive affective responses, which might improve their cognitive responses [24,26]. Although the evidence suggests the benefits of social engagement in PA as one of the main reasons for children’s participation [94], our study limited their social interaction, focusing only on the effects of the CDPA on their EF and enjoyment. The children overall enjoyed both conditions and reported it as being fun and enjoyable. These results might help inform further research and recommend how to design better a CDPA protocol that is more adapted to children’s preferences.

Among the strengths of this study, these protocols were matched for intensity and duration, and they involved no social interaction, whereby only the cognitive demands were manipulated. These conditions through video or verbal instruction can be easily implemented in various contexts such as schools, home-based interventions or outdoors by practitioners with little to no experience. Despite this, some limitations should be considered. Firstly, we did not control for social economic status differences, and it was not feasible to include a control condition due to the participants’ and parents’ burdens. Secondly, the physiological and anthropometric measures of intensity were unreliable to collect online. Thirdly, the depth of the interviews was limited due to the online nature of this study. Storyboarding might be recommended when conducting focus groups online to obtain a greater depth of responses. Fourthly, two participants underwent a one-on-one interview due to difficulty finding a slot suitable to children’s availability. Lastly, the participants spent a significant amount of time at home during the COVID-19 lockdown, which might justify their preference for outdoor activities.

## 5. Conclusions 

Our findings suggest that the acute effects of CDPA on the inhibitory and affective responses do not differ from those achieved with SPA. SPA and CDPA might be used in contexts to increase PA, wellbeing, build resilience and contribute to better mental health, and they are worth exploring further on other aspects of cognitive performance as academic performance can be consequently impacted. The children’s suggestions on changing these activities to make them more enjoyable using outdoor spaces and group and team games might be a key recommendation to practitioners and researchers who design similar activities. 

## Figures and Tables

**Table 1:** Interview guide, prompts and framework by Kallio et al. [76].

Area of Interest	Interview Guide	Prompts	Framework
**Introduction** (Ice-breaker)	Could you define the activities (SPA and CDPA) for me? What do you think these activities are for?		Identifying the prerequisites for using semi-structured interviews; Retrieving and using previous knowledge; Formulating the preliminary semi-structured interview guide; Pilot testing the guide; Presenting the complete semi-structured interview guide.
**Enjoyment** **Perception**	What did you like or dislike about it? What are your feelings about these activities?	Why did you like/dislike it? (e.g., intensity, duration, social aspects?) Did these feelings change before and after the activity? For how long did these feelings last?	
**Enjoyment** **Reflection**	How could these activities be changed for you and your colleagues to be more enjoyable or interesting? How would you design these activities?	What would make the activity more interesting? How could you make it more enjoyable? Participants are encouraged to think and/or draw activities	

**Table 2:** Cognitive performance scores for the Stroop test in ms (M ± SD).

	SPA			CDPA			Results		
	Baseline	1 min Post	30 min Post	Baseline	1 min Post	30 min Post	Condition	Time	Time * Condition
**Stroop test**									
Congruent (ms)	864 ± 185	807 ± 148	810 ± 147	849 ± 167	846 ± 172	818 ± 162	*p* = 0.23 η^2^p = 0.04	*p* = 0.43: η^2^p = 0.017	*p* = 0.33 η^2^p = 0.025
Congruent Accuracy ^a^ (%)	10 ± 12 *	7 ± 12 *	5 ± 7 *	11 ± 14	11 ± 14	13 ± 14	*p* = 0.046 * η^2^p = 0.103	*p* =.72: η^2^p = 0.004	*p* = 0.19 η^2^p = 0.046
Incongruent (ms)	925 ± 158	877 ± 168	878 ± 136	943 ± 179	911 ± 230	904 ± 190	*p* = 0.53 η^2^p = 0.01	*p* = 0.73: η^2^p=0.003	*p* = 0.65 η^2^p=0.006
Incongruent Accuracy ^a^ (%)	14 ± 14	13 ± 12	12 ± 13	17 ± 17	20 ± 19	18 ± 17	*p* = 0.38 η^2^p = 0.02	*p* =0.06: η^2^p = 0.09	*p* = 0.33 η^2^p = 0.03
Interference (ms)	60 ± 116	70 ± 88	68 ± 116	94 ± 92	65 ± 168	86 ± 125	*p* = 0.34 η^2^p = 0.024	*p* = 0.81 η^2^p = 0.005	*p* = 0.56 η^2^p = 0.015

* Significant at *p <* 0.05. ^a^ Represented by the percentage of incorrect responses (note that a lower value represents a better performance).

**Table 3:** The affect scales (feeling scale (FS) and felt arousal scale (FAS)), OMNI and mental exertion for SPA and CDPA are represented by medians and interquartile ranges (25% and 75% percentile).

		Baseline	Mid-Trial 1	Mid-Trial 2	1 Min Post	30 Min Post
**FS** **(Range −5 to +5)**	SPA	5 (3–5)	5 (4–5)	5 (3–5)	5 (3–5)	5 (5–5)
	CDPA	4 (3–5)	4 (3–5)	3 (2–5)	5 (3–5)	5 (3–5)
**FAS** **(Range 1 to 6)**	SPA	2 (1–3)	5 (3–5)	4 (3–6)	5 (4–6)	3 (2–5)
	CDPA	2 (1–2)	4 (3–5)	4 (3–6)	4 (4–6)	4 (2–6)
**OMNI** **(Range 0 to 10)**	SPA	2 (0–2)	3 (2–5)	4 (3–5)	6 (3–6)	2 (0–2)
	CDPA	1 (0–2)	3 (2–6)	4 (3–6)	5 (3–6)	2 (1–4)
**Mental exertion** **(Range 0 to 10)**	SPA	–	1 (0–2)	1 (0–2)	0 (0–2)	–
	CDPA	-	*3 (3–6)	*4 (3–9)	*4 (3–7)	-

* Significant at *p* < 0.05 (between conditions). Mid-trial 1 = following exercises 1 and 2, mid-trial 2 = following exercises 3 and 4 and 1 min post following exercises 5 and 6.

## Data Availability

Data supporting the results of the current study are available on request to the corresponding author.

## Appendix A

Adapted from PEBL and coded into Psytoolkit.

*“You are about to take part in one study in which you will be asked to determine the colour that written words appear in. Sometimes, the words will be actual colour names. When this happens, try not to respond with the written colour name, but only with the colour of the word. You will need to respond with the 1-2-3-4 keys on the top of the keyboard.”*

**Table A1:** Fidelity data for SPA and CDPA, represented for accuracy and number of movements described (range from 0–30) by raw data and percentage (for easier interpretation). Raw data were used for the analyses. * Significant *p <* 0.05.

Condition	SPA	CDPA	*t*-Test *p*-Value
**Accuracy of the movements**	28.2 ± 7 (94%)	26.1 ± 9 (87%)	0.004 *
**Number of repetitions**	28.2 ± 5 (94%)	27.8 ± 7 (92.6%)	0.333
